# Application of a pharmacokinetics-pharmacodynamics approach to the free propofol plasma levels during coronary artery bypass grafting surgery with hypothermic cardiopulmonary bypass

**DOI:** 10.6061/clinics/2018/e178

**Published:** 2018-01-08

**Authors:** Carlos R. Silva-Filho, Ricardo Antonio G. Barbosa, Carlindo V. Silva-Jr, Luiz M.S. Malbouisson, Maria José C. Carmona, Silvia Regina C. Jorge-Santos

**Affiliations:** I Faculdade de Ciencias Farmaceuticas, Universidade de Sao Paulo, Sao Paulo, SP, BR; II Servico de Anestesiologia e Terapia Intensiva Cirurgica Instituto do Coracao (InCor), Hospital das Clinicas HCFMUSP Faculdade de Medicina, Universidade de Sao Paulo, Sao Paulo, SP, BR

**Keywords:** Coronary Artery Bypass, Cardiopulmonary Bypass, Propofol, Protein Binding, Pharmacokinetics, Pharmacodynamics

## Abstract

**OBJECTIVES:**

The objective of this study was to apply a pharmacokinetics-pharmacodynamics approach to investigate the free propofol plasma levels in patients undergoing coronary artery bypass grafting under hypothermic conditions compared with the off-pump procedure.

**METHODS:**

Nineteen patients scheduled for on-pump coronary artery bypass grafting under hypothermic conditions (n=10) or the equivalent off-pump surgery (n=9) were anesthetized with sufentanil and propofol target-controlled infusion (2 μg/mL) during surgery. The propofol concentration was then reduced to 1 μg/mL, and a pharmacokinetics-pharmacodynamics analysis using the maximum-effect-sigmoid model obtained by plotting the bispectral index values against the free propofol plasma levels was performed.

**RESULTS:**

Significant increases (two- to five-fold) in the free propofol plasma levels were observed in the patients subjected to coronary artery bypass grafting under hypothermic conditions. The pharmacokinetics of propofol varied according to the free drug levels in the hypothermic on-pump group *versus* the off-pump group. After hypothermic coronary artery bypass was initiated, the distribution volume increased, and the distribution half-life was prolonged. Propofol target-controlled infusion was discontinued when orotracheal extubation was indicated, and the time to patient extubation was significantly higher in the hypothermic on-pump group than in the off-pump group (459 *versus* 273 min, *p*=0.0048).

**CONCLUSIONS:**

The orotracheal intubation time was significantly longer in the hypothermic on-pump group than in the off-pump group. Additionally, residual hypnosis was identified through the pharmacokinetics-pharmacodynamics approach based on decreases in drug plasma protein binding in the hypothermic on-pump group, which could explain the increased hypnosis observed with this drug in this group of patients.

## INTRODUCTION

Propofol is a hypnotic agent that is widely used in surgery because of its interesting pharmacokinetic (PK) properties. Target-controlled infusion (TCI) facilitates early orotracheal extubation after surgery, which might contribute to reducing the postoperative mechanical ventilation period, shortening patients' stays in the intensive care unit, lowering the incidence of nosocomial infections, and reducing hospital costs 1-3.

During coronary artery bypass grafting (CABG) surgery with cardiopulmonary bypass (CPB), profound changes in the effect of propofol and its kinetic behavior were observed based on total drug plasma measurements in these patients. As reported previously, drug plasma binding might be altered due to hemodilution after normothermic CPB because the free propofol plasma levels were increased 4-6. In addition, TCI is recommended for maintenance of the propofol plasma concentration (2 μg/mL) during CABG-CPB because its drug effect is rapidly lost in the liver, mainly through the CYP2B6 pathway. This pathway produces 2,6 di-isopropylquinol and 2,6 di-isopropyl-1,4-quinol derivatives, which are conjugated and excreted in the urine as quinol-1-glycuronide, quinol-4-glycuronide, and quinol-4-sulfate 7.

However, whether the significant changes in propofol hypnosis that occur in these patients are related to reduction of the extension of drug plasma binding remains unclear 8. Therefore, the objective of this study was to investigate the free propofol plasma levels in patients undergoing CABG with and without CPB using a PK-pharmacodynamics (PD) approach.

## MATERIALS AND METHODS

Nineteen patients were scheduled for elective cardiac surgery. The characteristics of these patients, expressed as the medians (quartiles), were as follows: age of 67.0 (61.5-69.5) years, weight of 73.7 (65.5-80) kg, and a body mass index (BMI) of 27.50 (25.12-29.42) kg/m^2^. The inclusion criteria were a left ventricular ejection fraction exceeding 50% and normal hepatic and renal function. The patients included in the study were stratified into two groups based on the surgeon's criteria as follows: Group 1, on-pump CABG with hypothermic (32-34°C) CPB (CPB-H, n=10); and Group 2, off-pump CABG (OPCAB, n=09). During the transoperative period, pre-anesthetic medication (midazolam: 0.2 mg/kg) was administered orally in the operating room, and a routine surgical protocol (previously detailed by the same authors) was then performed for each patient investigated 8. During the surgical intervention, hypnosis was achieved with propofol infusion to maintain a bispectral index (BIS) value of 40 based on a predicted plasma concentration of 2 μg/mL. At the end of surgery, the predicted drug plasma level was reduced to 1 μg/mL (Diprifusor^TM^, Astra-Zeneca, Detroit, MI, USA). During the intervention, hypnosis and hemodynamic parameters were monitored by anesthesiologists in the operating room throughout the transoperative period.

During the period from admission to the operating room until 12 hours after tracheal extubation, 3-mL blood samples were collected for drug plasma measurements, and the BIS was continuously monitored using a BIS^R^ XP device (Aspect Medical Systems, Natick, MA, USA) for brain activity measurements. The criteria for tracheal extubation were based on the routine protocol of our hospital as described previously by the same authors 8.

The blood samples were centrifuged to obtain plasma for drug measurements, and the samples were stored in a deep freezer (-80°C) until use. The propofol plasma samples were analyzed using a validated bioanalytical method based on high-performance liquid chromatography with fluorescence detection (HPLC-FD), as reported previously 7 for a two-compartment open model PK study – Non-Compartmental PK Data Analysis (Summit, USA). Ultrafiltration using Amicon-Ultra^®^ (Millipore, Ireland) was applied to separate bonded and free propofol from plasma samples. After purification of the biological matrix, the drug plasma level was measured using the LC10 fluorescence detector RF10AXL^®^ (276/310 nm) (Shimazdu, Kyoto, Japan). The chromatographic conditions consisted of a reverse phase column (ShimPack^®^ CLC – ODS C18 150 x 6.0 mm, 5 microns, Shimazdu, Kyoto, Japan) and a binary mobile phase of acetonitrile and water acidified with acetic to a pH of 4.6 (60:40, v/v). The mobile phase was prepared daily, degassed under helium (99.9%) strain and pumped isocratically at 0.8 ml/min, and each run time was 25 min. This method demonstrated linearity from 10 to 10.000 ng/mL, with a limit of detection of 5 ng/mL and a linear regression coefficient of 0.9977.

The estimated PK parameters were hybrid rate constants for drug distribution (α) and drug elimination (β) and their respective half-lives (t_(1/2)_α and t_(1/2)β_). Non-compartmental PK parameters, including drug plasma clearance (CL_T_) and the apparent volume of distribution (VDSS), were estimated by the area method.

In addition, PD was investigated by plotting the BIS values *versus* time, and a PK/PD approach was implemented based on a plot of the BIS values against the free propofol plasma levels over time. The sigmoid E_MAX_ model (variable slope) was selected to represent the hypnotic effect of propofol expressed by the BIS values as a function of the free drug plasma level.

A nonparametric statistical analysis was performed based on the Mann-Whitney U test, with the data expressed as the medians (quartiles). *P*-values <0.05 were considered statistically significant, and Prism v.5.0 software was used (Inc., San Diego, CA, USA).

### Ethics

The study protocol was approved by the Hospital Ethics Committee, and written informed consent was obtained from all included patients. All procedures were in accordance with good clinical practices and Brazilian regulations.

## RESULTS

Table 1 summarizes the demographic data of the individual patients. The data regarding age, body weight, BMI, and the duration of surgery were similar, as demonstrated through the group comparison, whereas the time required for orotracheal intubation differed significantly between the groups. The duration of surgery was similar between the patients subjected to either CPB-H (265 min) or OPCAB (260 min, *p*=0.7747). In contrast, the time to extubation was significantly longer for the patients receiving CPB-H (459 [301-526] min) than for the patients undergoing OPCAB (273 [198-289] min) (*p*=0.0048; median [quartiles]). The duration of the CPB procedure was 79 (64-95) min (median [quartiles]).

A total of 994 blood samples were analyzed to obtain the total and free plasma levels of propofol in the CPB-H (580 samples) and OPCAB (414 samples) patients. The median infused doses in both groups were comparable: 1510 mg (CPB-H) *versus* 1555 mg (OPCAB); *p*=0.2428. The total and free plasma levels of propofol did not differ between the groups throughout the study, as indicated in Table 2.

The PK of propofol based on the free plasma levels over time analyzed using a two-compartment open model are described in Table 3.

**Figure 1:**
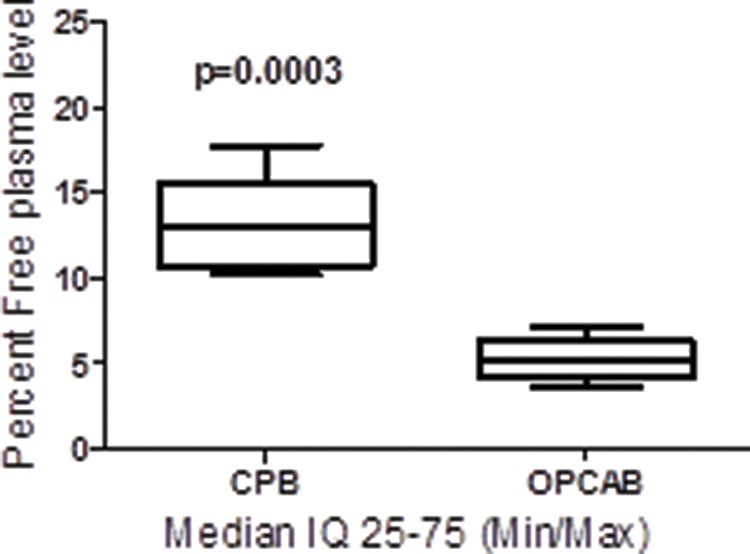
Increases in free drug plasma levels by hypothermic-CPB (medians [quartiles, min/max values]). **Abbreviations** – CPB-H: on-pump coronary artery bypass grafting; OPCAB: off-pump coronary artery bypass grafting. (▪) CPB-H, n=10; (□) OPCAB, n=9. **Statistics:** Mann-Whitney test, GraphPad Prism v. 5.0, significance *p*<0.05.

A comparison of the parameters of the two groups revealed significant changes in the PK of propofol: the plasma clearance and VDSS were increased in the CPB-H *versus* OPCAB groups due to reduced drug plasma binding caused by CPB-H. Consequently, the free propofol plasma levels of the CPB-H patients were increased relative to those of the OPCAB patients (Figure 1).

**Figure 2:**
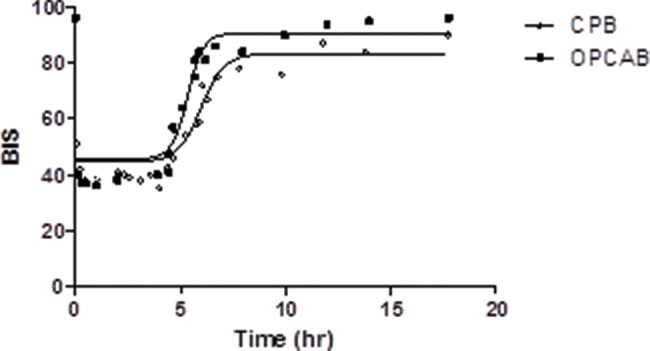
Pharmacodynamics modeling using the sigmoid E_MAX_ model. BIS values *versus* free propofol plasma levels (medians). **Abbreviations** – BIS: Bispectral index; CPB: on-pump coronary artery bypass grafting; OPCAB: off-pump coronary artery bypass grafting. (▪) CPB, n=10; (□) OPCAB, n=9. GraphPad Prism v. 5.0.

A PD approach based on measuring the drug effect over time indicated that during the intra-operative period, BIS≤40 was achieved, and the data were comparable between the groups. In contrast, after the drug infusion was reduced at the end of surgery and subsequently discontinued, the BIS values of the CPB-H patients were significantly lower than those of the OPCAB patients (Figure 2).

A PK/PD approach based on plotting BIS values against free drug plasma levels indicated that during the postoperative period, hypnosis in the CPB-H patients decreased more slowly than that in the OPCAB patients due to an increase in the free drug available to the receptor sites.

**Figure 3:**
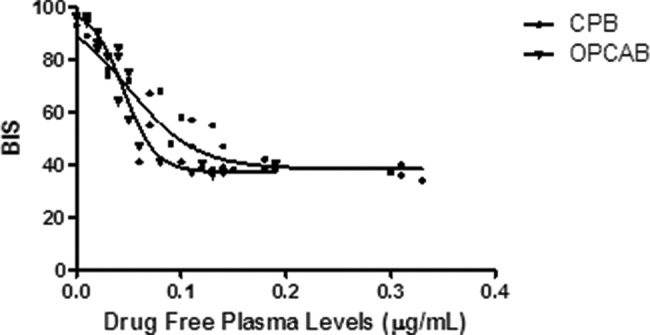
PK/PD analysis based on the sigmoid E_MAX_ model in the CPB-H group compared to the OPCAB group. The sigmoid shape was generated using the medians. BIS axis: Y_0_=85 in the CPB-H group *versus* Y_0_=95 in the OPCAB group at orotracheal extubation; EC_50_=1.12, r^2^>0.90. **Abbreviations** – BIS: bispectral index; CPB: on-pump coronary artery bypass grafting; OPCAB: off-pump coronary artery bypass grafting.

Although the propofol effect (expressed as the effective free drug concentration required to attain 50% of the maximum effect [EC_50_]) was comparable in both groups, the PK/PD approach could explain the differences between the groups based on the prolonged time to orotracheal intubation and awakening among CPB-H patients relative to OPCAB patients (Figure 3).

## DISCUSSION

The bioanalytical method was appropriate for propofol plasma measurements, particularly because it presented a wide range of linearity and a high coefficient of linear regression, illustrating the reliability of the method. A wide range of linearity was crucial for monitoring both the peak propofol levels during infusion and the extremely low concentrations after cessation of the TCI. In fact, HPLC with fluorescence detection is commonly reported as a technique of choice for the quantitative determination of propofol 9. Furthermore, ultrafiltration was an important addition to this method, which ultimately enabled separation of the free and protein-bound propofol in plasma 9,10.

The total propofol plasma levels of both groups were comparable during the surgical intervention. However, significant increases in the free propofol plasma concentration (two- to five-fold) were observed in the CPB-H group due to the CPB-H-induced reduction in drug plasma protein binding in these patients.

Our data are consistent with the results of previous reports and revealed that the unbound fraction of propofol in the blood increased two-fold during CPB 5. The authors of these previous reports stated that the total concentration of propofol in the blood remained unchanged after the initiation of normothermic CPB compared with the pre-CPB drug plasma concentrations. However, the fraction of unbound propofol in the blood doubled during CPB-H.

The decrease in propofol binding to plasma proteins that occurred after the initiation of CPB-H resulted in a significant increase in the fraction of unbound drug in the blood. Similar studies have also shown that CPB increases the plasma clearance of anesthetics, mainly due to alteration of the plasma protein binding of these drugs with albumin and alpha-1 glycoprotein acid 11-13. Propofol mostly binds to albumin and erythrocyte membranes 14. Hemodilution during CPB decreases the concentrations of albumin and hematocrit 4,6,15, leading to an increase in the free fraction of propofol under CPB-H. Nevertheless, it is assumed that the pharmacological effect of propofol ultimately reflects its unbound concentration because only drug that is not bound to plasma proteins can pass through membranes and reach the effect site 4. Therefore, the higher concentration of unbound propofol could explain the significantly lower BIS values observed in CPB-H patients after TCI cessation (i.e., greater effect) compared with those found in OPCAB patients. These observations are consistent with those previously reported in the literature 4-6,9,15,16.

The PK of free propofol determined based on the two-compartment open model was significantly altered in the CPB-H group compared with the OPCAB group due to the prolonged distribution half-life and the increased VDSS.

Drug plasma clearance was significantly higher in the CPB-H group due to the hypothermia associated with CPB-H, which is supported by the literature 9,. Propofol is known to be a high-extraction drug, and if drug plasma binding is reduced, the removal of propofol from the systemic circulation by the liver can be directly affected. Consequently, in the CPB group, plasma clearance increased due to heparinization and hemodilution in combination with CPB-H.

In addition, the kinetic behavior of propofol changes differently according to the free drug levels in the CPB-H group compared with those in the OPCAB group. These results are consistent with those of Peeters et al. (2008) 17 and Bienert et al. (2010) 18, who investigated the PK of propofol in 26 and 28 critical patients, respectively, using the two-compartment model with NONMEN software. Subsequently, Wiczling et al. (2012) 19 also demonstrated the adequacy of the two-compartment model in a study of the PK of propofol in 10 patients during elective abdominal aortic surgery.

Regarding the PK/PD approach, the selected sigmoid E_MAX_ model (variable slope) revealed a strong correlation between the BIS values and free drug levels (r^2^>0.90, *p*<0.001) in both groups.

Despite the similar total drug plasma levels after propofol infusion cessation, the BIS values in the CPB-H patients remained lower than those in the OPCAB patients. In fact, the time to orotracheal intubation was significantly longer in the CPB-H group than in the OPCAB group (459 min *versus* 273 min, *p*<0.001) despite similar surgical durations (265 min for the CPB-H group *versus* 260 min for the OPCAB group; *p*=0.7747). These data are consistent with the reported results of increases in the drug effect after CPB without changes in the total drug plasma levels 4,6.

Based on the results of our application of our PK/PD approach to the free propofol plasma levels, we propose that the important changes in the PK of propofol induced by CPB-H described above could explain the increased post-CPB hypnotic effects observed in patients undergoing CPB-H.

Additionally, the increased depressant effect on the central nervous system observed in the CPB-H group could be explained by the increased VDSS and the prolonged distribution half-life as a function of high levels of free propofol in the systemic circulation, which are associated with the high free drug concentrations at receptor sites.

In conclusion, the significant increases in the free propofol plasma concentration in the CPB group were attributed to the reduction in drug plasma protein binding resulting from CPB-H, which ultimately led to postoperative alterations in the PD of propofol.

## AUTHOR CONTRIBUTIONS

Silva-Filho CR was responsible for the data analysis, PK-PD modeling and manuscript writing. Barbosa RA administered anesthesia to the patients and performed the data collection. Silva-Jr CV contributed to the data analysis and manuscript writing. Malbouisson LM cared for the patients in the intensive care unit. Carmona MJ contributed to conducting the study and administering anesthesia. Jorge-Santos SR was responsible for the overall coordination of the study, sample analysis, data analysis, PK-PD modeling, and manuscript writing. All authors contributed to revising the manuscript.

## Figures and Tables

**Table 1:** Demographic characteristics of the individual patients and surgical data.

Patient allocation	Gender (M/F)	Age (yrs)	BW (kg)	BMI (kg/m^2^)	Duration of surgery (min)		Orotracheal intubation (min)	
					CPB-H	OPCAB	CPB-H	OPCAB
1	M	67	66.0	23.9	270		292	
2	M	64	92.5	34.4	230		287	
3	F	74	63.0	28.0		270		232
4	M	56	84.0	32.8	310		292	
5	F	67	78.0	27.4		330		238
6	M	59	65.0	22.8	235		534	
7	M	74	65.0	23.3	325		549	
8	F	52	71.0	33.3		260		198
9	F	69	76.0	26.5	225		519	
10	M	67	72.6	29.5	390		330	
11	M	68	76.2	27.7	300		450	
12	M	66	90.0	29.3		205		289
13	M	56	65.0	25.4	260		468	
14	M	49	90.0	35.2	230		528	
15	M	72	73.7	27.5		220		339
16	M	70	64.8	23.6		210		378
17	M	68	82.0	28.1		255		273
18	M	75	71.0	25.2		330		186
19	F	68	74.2	25.1		335		198
Median (quartiles)	N/A	67.0 (61.5-69.5)	73.7 (65.5-80.0)	27.50 (25.12-29.42)	265 (231-308)	260 (220-330)	459 (301-526)	273 (198-289)

**Patient allocation** - CPB-H group: 1, 2, 4, 6, 7, 9, 10, 11, 13, 14; OPCAB group: 3, 5, 8, 12, 15, 16, 17, 18, 19. **Abbreviations** – N/A: not applicable; BW: body weight; BMI: body mass index; F: female; M: male; CPB-H: on-pump coronary artery bypass grafting; OPCAB: off-pump coronary artery bypass grafting. **Statistics:** Mann-Whitney test, GraphPad Prism v. 5.0, significance *p*<0.05.

**Table 2:** Propofol plasma levels over time.

Time of sampling	Total propofol plasma levels (μg/mL), *p*>0.05		Free propofol plasma levels (ng/mL), *p*>0.05	
Starts TCI	CPB-H (n=10)	OPCAB (n=9)	CPB-H (n=10)	OPCAB (n=9)
(min)	Medians (quartiles)	Medians (quartiles)	Medians (quartiles)	Medians (quartiles)
5	1.77 (1.09-2.89)	1.79 (1.63-2.59)	94.87 (48.96-160.92)	96.17 (73.92-179.52)
15	1.39 (1.11-1.98)	1.71 (1.45-3.03)	59.36 (52.00-109.65)	95.70 (66.88-164.70)
30	2.37 (1.79-2.99)	2.75 (2.03-3.42)	140.76 (88.20-153.70)	147.40 (75.11-161.67)
60	1.94 (1.21-2.55)	1.60 (1.35-2.51)	125.82 (69.75-146.88)	86.97 (59.20-138.05)
120	2.43 (1.92-3.04)	3.15 (2.42-3.61)	141.34 (103.35-166.32)	142.78 (113.30-233.64)
Before CPB-H	2.20 (1.95-2.88)	N/A	109.62 (97.50-139.50)	N/A
5	1.88 (0.96-2.66)	N/A	118.19 (50.40-142.80)	N/A
15	2.18 (1.63-2.80)	N/A	144.72 (72.90-154.66)	N/A
30	2.25 (1.72-2.68)	N/A	134.09 (75.15-139.23)	N/A
60	2.81 (1.93-3.16)	N/A	71.55 (0.00-153.90)	N/A
End of CPB-H	2.07 (1.30-2.68)	N/A	141.51 (53.7-150.22)	N/A
240	1.22 (0.80-2.65)	1.72 (29-5.62)	46.98 (36.54-182.52)	80.10 (47.57-261.96)
End of Surgery	1.40 (0.51-1.69)	1.29 (1.23-1.92)	58.50 (17.28-92.88)	76.11 (55.35-85.17)
5	1.04 (0.83-1.27)	1.02 (0.92-1.26)	54.57 (42.66-67.00)	55.44 (37.74-65.32)
15	0.71 (0.47-27)	0.80 (0.65-1.16)	28.35 (22.26-58.14)	45.43 (35.20-47.85)
30	0.96 (0.61-1.19)	0.91 (0.80-0.97)	43.46 (31.32-69.30)	40.29 (37.83-52.80)
60	0.68 (0.45-0.95)	N/A	23.54 (17.08-46.73)	N/A
End of TCI	0.87 (0.63-1.12)	0.97 (0.76-1.27)	42.40 (30.09-50.76)	40.95 (37.00-64.77)
5	0.90 (0.64-0.97)	0.78 (0.68-0.86)	44.28 (23.97-48.06)	35.10 (30.81-44.88)
15	0.69 (0.50-0.77)	0.61 (0.45-0.75)	31.95 (19.89 - 38.88)	27.75 (21.83 - 40.26)
30	0.47 (0.37-0.65)	0.51 (0.37-0.59)	21.20 (13.77-29.25)	21.83 (17.11-31.11)
60	0.38 (0.24-0.56)	0.41 (0.33-0.54)	14.84 (9.72-26.55)	19.61 (15.34-30.09)
120	0.26 (0.17-0.41)	0.36 (0.30-0.48)	9.72 (8.16-17.82)	17.76 (13.50-23.46)
240	0.22 (0.12-0.31)	0.31 (0.22-0.37)	6.89 (5.10-13.95)	15.81 (9.45-19.25)
360	0.16 (0.09-0.25)	0.25 (0.19-0.34)	5.30 (4.08-8.64)	13.06 (7.93-17.82)
480	0.13 (0.08-0.17)	0.18 (0.14-0.29)	4.72 (3.17-7.52)	9.18 (6.30-12.65)
720	0.09 (0.05-0.11)	0.14 (0.11-0.22)	4.50 (2.39-5.36)	7.14 (4.84-11.00)

**Abbreviations** – TCI: target-controlled infusion; CPB-H: on-pump coronary artery bypass grafting; OPCAB: off-pump coronary artery bypass grafting; N/A: not applicable. **Statistics:** Mann-Whitney test, significance *p*<0.05.

**Table 3:** Pharmacokinetics of free propofol (medians [quartiles]).

PK- parameters		CPB-H	OPCAB	Statistics (*p*)
Alpha	(h-1)	2.20 (2.07-2.72)	3.89 (3.00-4.34)	0.0010
t_(1/2)α_	(h)	0.32 (0.26-0.34)	0.18 (0.16-0.23)	0.0019
Beta	(h-1)	0.13 (0.11-0.16)	0.114 (0.10-0.12)	0.2352
t_(1/2)β_	(h)	5.37 (4.34-6.38)	6.10 (5.74-6.78)	0.3270
VDSS	L/kg	14.45 (12.73-18.50)	10.2 (9.90-12.50)	0.0293
CL_T_	mL/min.kg	36.90 (32.77-39.12)	18.97 (14.94-21.38)	0.0070

**Abbreviations** – CPB: on-pump coronary artery bypass grafting-cardiopulmonary bypass; OPCAB: off-pump coronary artery bypass; N/A: not applicable. **Statistics:** Mann-Whitney test, GraphPad Prism v. 5.0, significance *p*<0.05.
